# To what extent has the Iranian Health Transformation Plan addressed inequality in healthcare financing in Iran?

**DOI:** 10.1186/s12939-023-01880-z

**Published:** 2023-04-06

**Authors:** Maryam Moeeni, Shirin Nosratnejad, Manizhe Rostampour, Koen Ponnet

**Affiliations:** 1 Social Determinants of Health Research Center, Isfahan University of Medical Sciences, Isfahan, Iran; 2 Iranian Center of Excellence in Health Services Management, School of Management and Medical Informatics, Tabriz University of Medical Sciences, Tabriz, Iran; 3 Faculty of Social Sciences, Imec-Mict-Ghent University, Ghent, Belgium

**Keywords:** Health financing system, Financing system, Equity in healthcare financing, Iran

## Abstract

**Background:**

One of the major goals of health systems is providing a financing strategy without inequality; this has a significant impact on people’s access to healthcare. The present study aimed to investigate the inequality in households’ financial contribution (HFC) to health expenditure both before and after the implementation of the Iranian Health Transformation Plan (HTP) in 2014.

**Methods:**

This study is a secondary analysis of two waves of a national survey conducted in Iran. The data were collected from the Households Income and Expenditure Survey in 2013 and 2015. The research sample included 76,195 Iranian households. The inequality in households’ financial contributions to the health system was assessed using the Gini coefficient, and the concentration index (CI). In addition, by using econometric modeling, the relationship between the implementation of the HTP and inequality in HFC was studied. The households’ financial contribution included healthcare and health insurance prepayments.

**Results:**

The Gini coefficient values were 0.67 and 0.65 in 2013 and 2015, respectively, indicating a medium degree of inequality in HFC in both years. The CI values were 0.54 and 0.56 in 2013 and 2015, respectively, suggesting that inequalities in HFC were in favor of higher income quintiles in the years before and after the implementation of the HTP. Regression analysis showed that households with a female head, with an unemployed head, or with a head having income without a job were contributing more to financing health expenditure. The presence of a household member over the age of 65 was associated with a higher level of HFC. The implementation of the HTP had a negative relationship with the HFC.

**Conclusion:**

The HTP, aiming to address inequality in the financing system, did not achieve the intended goal as expected. The implementation of the HTP neglected certain factors at the household level, such as the presence of family members older than the age of 65, a female household head, and unemployment. This resulted in a failure to reduce the inequality of the HFC. We suggest that, in the future, policymakers take into account factors at the household level to reduce inequality in the HFC.

## Background

Equity in the healthcare sector can be achieved by providing health services based on patients’ needs and their ability to pay. Therefore, a health system can be considered equitable when people with the same healthcare needs have access to the same or equal healthcare services and can participate in the payments equally [1], even if they live in different social and economic conditions. people’s access to healthcare services, and their fair contribution to expenditure, are influenced by the financial systems [2].

Out-of-pocket (OOP) payments in low- and middle-income countries account for a larger share of the financial system than other financing mechanisms [3], so that, according to a World Health Organization (WHO) report in 2019, household contribution to direct OOP payments varied from 47% in low-income countries to 34% in middle-income countries [4].

Financial constraints in households are a major reason for the lack of access to healthcare services [5, 6]. Low-income households commonly need more healthcare services than high-income ones [7]. In addition, these households are more affected by the high costs of healthcare services [8]. Thus, the reduction in the inequality of households’ financial contributions to healthcare expenditure is one of the most important goals for governments and health policymakers [9]. A fair health system attempts to provide all citizens with equal access to treatment and healthcare services, and protects people from the costs and financial risks of diseases [10, 11]. Some developing countries undertake relevant health reforms to reduce health inequalities [12]. In Iran, inequality in health expenditure is a serious concern. Direct OOP payments account for half the health expenditure of Iranian households, and nearly 6.97% of Iranian families are exposed to a catastrophic level of healthcare expenditure [13].

In recent years, several reforms have been implemented in the Iranian health system; unfortunately, because of both structural and nonstructural causes, most of these reforms have not been very successful in achieving equity in the health system [14]. In 2014, the Iranian government implemented a new reform plan for the healthcare system, the Iranian Health Transformation Plan (HTP) [15]. The HTP included actions aiming to offer financial protection against health expenditure [16], mainly by the reduction of OOP payments, and the provision of universal health coverage [17, 18]. In order to achieve these goals, the HTP took certain actions, such as providing free social health insurance for uninsured Iranian citizens, financially protecting patients suffering from severe and specific diseases, reducing OOP expenditures for inpatient services with a primary focus on the public hospitals affiliated with the Ministry of Health and Medical Education (MOH), and revising the medical tariffs to consider the relative value units of clinical procedures [15, 16].

As the HTP was an innovative plan in Iran’s health system, many studies have investigated different aspects of the plan. For instance, some studies have examined how the HTP affected health indicators, such as impoverishment of households [19], increasing health expenditure [20], utilization of health services, inpatient payments [21], and health impact indicators [22]. A few studies have relied on a large sample size at a national level [23], and several studies have evaluated small datasets at local levels [20, 22]. As expected, existing studies have also applied different research methods, and both qualitative [21] and quantitative approaches [24]. The present study aims to investigate the possible change in inequality in households’ financial contributions to healthcare expenditure, including direct and indirect expenditure, before and after the implementation of the HTP. Iran is still facing inequality struggles with economic stagnation and increasing inflation rates. Iranian households have difficulty affording health care costs [25]. By doing this study, we tried to learn from the past and provide health policy makers with better insights into the inequality in health care financing in Iran. It may provide new scientific information for making effective policies to alleviate inequality in health financing. To the best of our knowledge, the current study is the first one in Iran that attempts to study the role of the HTP on inequality in HFC to health expenditure based on two waves of a large national dataset.

## Methods

### Data and study population

The present study is a secondary analysis of two waves of the Households Income and Expenditure Survey (HIES), a national cross-sectional survey conducted annually in a representative sample of households in Iran. The data from the HIES in 2013 and 2015 were applied; these years were chosen as the years before and after the implementation of the HTP. The initial study sample included 38,316 rural/urban households in 2013 and 38,252 rural/urban households in 2015, collected from 31 provinces in Iran. Households with imperfect demographic and economic information were excluded from the study; thus, the final sample included 76,195 households. Total household expenditure data in the HIES includes the sum of the food and non-food expenditure of the households based on ten types of costs recorded in the survey questionnaire. Health expenditures (OOP payments), and insurance prepayments were included among the costs recorded for the last month prior to the survey date. In the current study, household expenditure was adjusted based on the total consumer price index (CPI), and health expenditure was adjusted based on the price index of the health sector compared to the base period of 2011.

HFC to health expenditure, was the outcome variable. Main component of the outcome variable was health expenditure. To more detail, HFC to health expenditure was measured by the deviation of health expenditure to household capacity to pay. Thus, HFC to health expenditure was calculated as follows:

$$HFC= \frac{HE}{CTP}= \frac{H{E}_{i}}{ (exp-food)}$$

In the above equation, *“HE”* is the total health expenditure of each household, which includes social and private health insurance prepayments plus direct OOP payments for health services. *“CTP”* is household capacity to pay, which indicates the effective income above the minimum subsistence (living) level. In addition, *“exp”* is the total expenditure of each household. Since, the information on household income was not accurate, the household total expenditure was taken as an index for the income level. The *“food”* here is household food expenditure, which is assumed to be the minimum subsistence (living) index.

### Data analysis

In the first step, we applied Gini index to measure and compare the distribution of HFC to health expenditure across the samples in 2013 and 2015, the years before and after implementation of the HTP. The Gini coefficient is a measure of statistical dispersion which is useful for quantifying inequalities within a population [26]. This coefficient is based on Lorenz curves ranging from 0 to 1. A Gini coefficient of zero means perfect equality in the health variable (in this case, HFC), where values are the same for all, and the Gini coefficient closer to 1 indicates greater inequality within a population.

Next, we measured and compared income inequality in HFC to health expenditure before and after implementation of the HTP based on the concentration index (CI). CI is a relative measure of inequality commonly used to summarize health inequality between subgroups with ordinal ranking [26]. The magnitude of the concentration index ranges from -1 to 1. The negative values indicate the health variable is concentrated among disadvantaged groups, while the positive values indicate the health variable is concentrated among advantaged groups. When there is no inequality, the concentration index is 0 [26]. In the present study, the ranking variable for estimating CI was the household’s income quintile. We applied the HIES data on weighted gross cost decile at province level to construct a proxy variable for income quintile. Based on weighted gross cost decile, the sample households were allocated into four income quintiles, including 1) households with the lowest income (the first income decile), 2) low-income households (the second income decile), 3) middle-income households (the third income decile), and 4) high-income households (the fourth income decile).

In the final step, data from the HIES in 2013 and 2015, as the years before and after implementation of the reform plan, were combined as pooled data. Then, econometric modeling was applied to investigate the relationship between the implementation of the HTP, the main independent variable, and the household’s financial contribution to healthcare expenditure in Iran. The other independent variables included demographic variables of the household’s head, economic variable, and health-related variables.

The regression model used the following formula:

$$Y= {\beta }_{0}+ \beta X+u$$

In the above formula, X includes independent variables, and Y is the HFC.

The independent variables were included in the model according to a forward stepwise approach. For this purpose, first the socio-demographic variables of the household’s head, that is, age, gender, employment status, marital status, and educational level, as well as number of family members, were included in the regression model, and subsequently health-related variables, including the presence of individuals in the household aged younger than five, and the presence of individuals older than the age of 65, were added. Finally, the variable of reform plan implementation was added to the model.

## Results

The descriptive results of the 2013 sample indicated that about 88% of the household heads were male, and 87% were married. In terms of employment, 71% were employed, and 40% had elementary education levels. The average household size in the 2013 sample was 3.63 members, and the average age of the household heads was 48.5 years. Regarding the 2015 sample, around 86% of the household heads were male, and nearly 85% were married. In addition, about 67% were employed, and 38% had elementary education levels. The average household size in the 2015 sample was 3.59, and the average age of the household heads was 50.6 years.

Table 1 presents the descriptive statistics of HE and HFC to health expenditure in total sample households, and across income quintiles. On average, both HE and HFC to health expenditure were higher for households in upper income quintiles.

**Table 1:** Descriptive statistics of HFC

Income quintile	HE & HFC to health expenditure			
	2013		2015	
	HE in PPP^a^ int. $ (SD)	HFC	HE in PPP^a^ int. $ (SD)	HFC
First quintile	4.12 (2.06)	0.01	5.3507 (3.69)	0.01
Second quintile	11.72 (4.01)	0.02	16.09 (7.50)	0.03
Third quintile	24.29 (9.78)	0.04	36.03 (18.49)	0.05
Fourth quintile	115.84 (150.30)	0.11	142.1 (232.69)	0.10

^a^ Purchasing Power Parity

The results of the Gini coefficient and CI are reported in Table 2. The Gini coefficient values in 2013 and 2015 were equal to 0.67 and 0.65 (*P*_value < 0.05), respectively. Gini coefficient was a measure of statistical dispersion represented distribution of HFC to health expenditure across each sample of our study. The calculated Gini values represented unequal distribution of HFC to health expenditure within each sample. However, Gini values of two samples were fairly the same indicating very similar degrees of inequality in HFC to health expenditure in the years of 2013 and 2015; the fairly similar degrees of inequality in HFC to health expenditure were found within the sample households in both year before implementation of HTP and year after that.

**Table 2:** Gini coefficient and CI values for HFC

Year	Gini coefficient value	*p*-value	CI value	*p*-value
2013	0.67	0.000	0.54	0.000
2015	0.65	0.000	0.56	0.000

Although, calculated values of Gini coefficient declared the unequal distribution of HFC to health expenditure within total sample of households before and after implementation of the HTP, they did not provide any information about distribution of HFC to health expenditure between the income subgroups. Subsequent measurement of CI values yielded a good way to analyze distribution of HFC to health expenditure between the income deciles. The CI values in 2013 and 2015 were equal to 0.54 and 0.56 (*P*_value < 0.05), respectively. The degree of inequality in HFC to health expenditure was not reduced in 2015; in fact, it increased very slightly by 0.02. Thus, Comparison of the CI values estimated for 2013 and 2015 indicates that inequalities in the HFC to health expenditure were in favor of higher income quintiles in both year before implementation of HTP and year after that. However, the CIs was fairly similar meaning that income inequality in HFC to health expenditure did not changed considerably after implementation of HTP.

The results obtained from estimating the ordinary least square (OLS) regression models, for household data, are presented in stepwise mode in Table 3. To investigate the relationship between the potential variables and the HFC to health expenditure, the sign of the coefficients and the significance level were assessed.

**Table 3:** Results of regression model for pool data at household level

variable	Model 1	Model 2	Model 3
Family size	-0.214^a^	-0.175^a^	-0.172^a^
Age	-0.0162^a^	-0.0224^a^	-0.0198^a^
Gender (ref = male)	-	-	-
Female	0.633^a^	0.774^a^	0.735^a^
Employment status (ref = employed)	-	-	-
Unemployed	1.912^a^	1.777^a^	1.798^a^
Has an income without a job	1.122^a^	1.001^a^	0.967^a^
Marital status (ref = married)	-	-	-
unmarried	-0.205	-0.42	-0.37
Educational level (ref = Primary)	-	-	-
Middle and high school	-0.541^a^	-0.471^a^	-0.424^a^
Diploma and post-diploma	-1.472^a^	-1.252^a^	-0.837^a^
academic education	-0.436^a^	-0.252^a^	-0.534^a^
Informal training	-0.199	-0.319	-0.035
A household member under the age of 5	-	-0.036	-0.031
A household member over the age of 65	-	0. 956^a^	0.930^a^
Implementation of the HTP			-1.129^a^

^a^ Significance at the 5% level

According to the first model, the following variables were significant: household size, age, gender, employment status and educational level. In particular, household size, and household head’s educational levels of middle school, high school, diploma, and academic degree were negatively associated with the HFC to health expenditure. Moreover, as the age of the household head rose, the HFC to health expenditure decreased very slowly. However, those households with a female head, with an unemployed head, and with a head having income without a job were contributing more to financing health expenditure. In the second model, health related variables were added. The presence of a household member younger than the age of five had no significant relationship with the HFC to health expenditure. However, having a family member older than the age of 65 was statistically significant, and the positive sign of its coefficient indicated its association with the HFC to health expenditure. In other words, when there were individuals older than the age of 65 in the household, the HFC to health expenditure increased. In the final model, the variable of reform plan implementation was added. The implementation of the HTP was significantly and negatively associated with the reduction in HFC, although the association was rather low.

## Discussion

The present study aimed to investigate the association of the implementation of the HTP with the degree of inequality in HFC. The results suggest that implementation of this reform plan had no great association with the reduction of inequality in the household contributions to healthcare financing.

The Gini coefficient and CI values were 0.67 and .54 in 2013, and 0.65 and .56 in 2015, respectively, indicating a very similar degree of inequality in the household contributions to healthcare financing across the studied years. In other words, after the implementation of the reform plan, inequality did not decrease in any considerable way. In two similar studies, Abdi et al. (2020) used data from the HIES in 2014 and 2015 to calculate the Kakwani index for households’ OOP. The results indicated that OOP spending became slightly more progressive over the time period of HTP reform [27], and Homaie Rad et al. (2017) calculated concentration indices for outpatient and inpatient expenditure, as well as drug services expenditure in Guilan Province before and during the HTP reform using data from the HIES for the years 2013 and 2015. The findings suggested that inequality in OOP payments did not significantly change after the HTP reform in the local sample of Guilan province [22]. These two research studies measured the change in inequality in somewhat different categories of health spending, and the later study was conducted in a local sample. Still, their findings support the notion that HTP reform may not decrease inequality in household spending for healthcare services.

The econometric model used in the present study revealed that, holding other variables constant, after the implementation of the HTP, households’ financial contribution to healthcare increased slowly. Thus, the reform plan may not have achieved the intended goals of reduction of households’ contribution to healthcare expenditure. It seems that the reform plan paid little attention to household factors influencing the HFC to health expenditure. As a result, the general approach of the plan, based merely on financial transformation, may not have played an effective role in the reduction of inequality in HFC. Therefore, in the current study, the potential household correlates of HFC to health expenditure are included in the regression models as control variables. As expected, the finding confirmed the significant correlation between these households’ factors with the HFC to healthcare expenditure.

Socio-demographic variables were the first category of controls. We found that households with more members, those with a better-educated head, and with an older head had, on average, lower contributions to healthcare expenditure. Existing literature supports these findings. Alizadeh et al. (2002) applied the econometric method to study the correlates of HFC to health expenditure in Iran. The findings suggested that a larger family size, employment status of the household head, and her/his education level reduced the HFC to health expenditure [28]. However, inconsistent with current work, Alizadeh et al.’s (2002) study reported that the increased age of the households’ heads resulted in a higher contribution of the households to healthcare expenditure. Regarding demographic correlates, we also found the femaleness of the household head and the presence of a family member older than the age of 65 led to an increased HFC to the health expenditure. Health variables were the other category of potential correlates included in regression models. Among them, the presence of family members older than the age of 65 related to an increase in the average financial contribution of the household to the healthcare expenditure.

Gender of the household head, and age structure of its members, are important for policymaking. Households with a female head are at higher risk of economic vulnerability. Moreover, older citizens generally require more healthcare services; this can cause an increase in healthcare expenditure among households with an elderly member. These findings imply that, although the HFC to health expenditure is concentrated among the rich, it is families who are vulnerable, aside from their income situations, who have paid a higher proportion of their capacity to pay for healthcare services, both before and after the introduction of the HTP. As a result, vulnerable groups may incur unaffordable health expenditure. Therefore, in order to achieve effective policymaking, health system reforms should prioritize the healthcare needs of these particular households. In line with this finding, Ramezanian et al. (2020) confirmed that vulnerable social classes incurred a higher burden of the HFC to health expenditure [29].

The current period is financially a difficult period for Iranian people, because there is a financial crisis, economic growth is slow, and inflation rates are high [30]. As a result, Iranian people mainly vulnerable households face much more difficulties paying for heath cares. The result of current study brings better understanding about inequality in health financing in this hard economic situation in Iran. It can be suggested that a good health financing reform provided by the government is necessary in this economic situation. That health financing reform should consider diversities in the demographic characteristics and health status of households.

### Limitation

The present study was subject to an especial limitation. Household income in national surveys can be subject to inaccuracies because it relies on self-reports. To mitigate this limitation in the current study, household total expenditure was taken as an index for the income level.

## Conclusion

The HTP, which aimed to address inequality in the health financing system, did not achieve the intended goal as expected. The implementation of the HTP neglected factors at the household level, such as the presence of family members older than the age of 65, a female household head, and unemployment. This resulted in a failure to reduce the inequality of the HFC. In conclusion, if purposeful health system reforms consider diversities in the demographic characteristics and health status of households, they can help to reduce inequality in Iranian households’ contribution to healthcare financing.

## Data Availability

Data are available from the authors upon reasonable request and with permission from the Statistical Center of Iran.

## Abbreviations

CI: Concentration index
CPI: Consumer price index
CTP: Household capacity to pay
HE: Health expenditure
HFC: Households’ financial contribution
HIES: Households Income and Expenditure Survey
HTP: Health Transformation Plan
MOH: Ministry of Health and Medical Education
OLS: Ordinary least square
OOP: Out-of-pocket
PPP: Purchasing Power Parity
WHO: World Health Organization
